# 3-dimensional plasmonic nanomotors enabled by independent integration of optical pulling and lateral forces

**DOI:** 10.1515/nanoph-2025-0374

**Published:** 2025-09-22

**Authors:** Guillermo Serrera, Yoshito Y. Tanaka, Pablo Albella

**Affiliations:** Group of Optics, Department of Applied Physics, University of Cantabria, 39005 Santander, Spain; Research Institute for Electronic Science, Hokkaido University, Kita21, Nishi 10, Kita-ku, Sapporo, Hokkaido 001-0021, Japan

**Keywords:** nanotechnology, nanophotonics, plasmonics, optical manipulation, optical forces, optical pulling

## Abstract

Light–matter interactions generally involve momentum exchange between incident photons and the target object giving rise to optical forces and torques. While typically weak, they become significant at the nanoscale, driving intense research interest in the exploitation of photon recoil to drive micro- and nanostructures. While great progress has been attained in controlling transversal degrees of freedom, three-dimensional movement remains challenging, particularly due to the impractical realization of pulling forces that oppose the direction of incident light. Here we theoretically present a novel nanomotor design that enables independent control over both transverse and longitudinal motion. This design exploits coupling between an azimuthally polarized Bessel beam and a dielectric glass cylinder to realistically achieve optical pulling forces. At the same time, asymmetric plasmonic dimers, embedded within the cylinder, provide lateral motion, through asymmetric scattering under plane wave illumination. We further demonstrate that unwanted displacements and rotations can be restrained, even at long illumination times. Our design unlocks a new degree of freedom in motion control, allowing for pulling, pushing, and lateral movement by simply tuning the polarization or switching between plane waves and Bessel beams.

## 1 Introduction

At the core of light–matter interaction, photons are scattered or absorbed by objects leading to an exchange or transfer of linear and angular momentum. This, by Newton’s third law, results in optical forces and torques acting back on the interacting objects [1], [2]. The ability to remotely manipulate objects has prompted intense research into microscopic light-driven actuators, looking for the miniaturization and simplification of current systems, with a focus on lab-on-a-chip technologies [3], [4]. These actuators often include fluid pumps [5], [6], valves [7], and mixers [8], as well as motion actuators fueled by strong gradient forces in tightly focused beams [9], [10]. However, reliance on gradient forces imposes some design constraints, including the need for beam steering, its short working range due to the low depth of focus of high NA lenses, and the limitations imposed by diffraction into the light intensity gradient. Most importantly, the interplay between gradient and scattering forces enforces severe limitations on the actuated object’s geometry and material.

All these constraints have motivated the study of scattering forces with plane waves or weakly focused beams as an alternative to optical tweezers and gradient-driven forces. In particular, the exploitation of asymmetric scattering from nanostructures has led to some successful lateral optical nanomotors. These schemes have relied on asymmetric plasmonic assemblies, such as gammadion shapes [11], dimers/trimers resembling Yagi–Uda antennas [12], [13], or dielectric metasurfaces with directional scattering [14]. Combinations of these elements allow for control of different degrees of freedom within the transversal plane, including both translation and rotation.

However, controlling longitudinal motions without gradient forces remains a challenge in this type of devices. ‘Pushing’ forces are a trivial byproduct of light scattering at normal incidences, arising as a direct result of momentum conservation. On the other hand, ‘pulling’ scattering forces are counter-intuitive, since $F_{\text{long}}=\frac{W_{\text{scat}}}{c}\left(\cos\theta_{0}-\left\langle \cos\theta \right\rangle \right)$ , where the longitudinal force *F*_long_ is given by the scattered power *W*_scat_ and the relationship between the cosine of the incident angle cos *θ*_0_ and the scattering angular average $\left\langle \cos\theta \right\rangle$ . This means that the optical pulling effect is attained when the object collimates the incident light ( $\left\langle \theta \right\rangle <\theta_{0}$ ) [15]. Since normally incident plane waves are characterized by *θ*_0_ = 0, they cannot sustain optical pulling forces. As a result, alternative methods must be considered, like the use of internal bubbles [16], topology-engineered materials [17], [18], interactions with interfaces [19], strong chiral light–matter interactions [20], [21], exploiting symmetry in Bound States in the Continuum [22] or complex optical conveyors [23], [24], [25].

Nonetheless, the two most successful approaches for realizing optical pulling forces have been the use of Bessel beams [26] and optical tractor beams (OTBs), based on the interference of several obliquely incident plane waves [27], [28]. In both cases, the individual photons exhibit oblique incidence angles *θ*_0_. While pulling forces have been successfully achieved using very large angle Bessel beams and OTBs, they are impractical due to their short range [29]. In this respect, azimuthally polarized Bessel beams, coupled to anti-reflective coated dielectric cylindrical waveguides have shown to be an effective approach to reduce angles below 45°, where range is no longer suppressed [30].

While these various techniques for optical pulling exist, their realistic application in the control of longitudinal motion in nanomotors is still missing, and current designs, based on sophisticated responses to different incident polarizations, are not suitable for this purpose.

In this work, we propose a realistic nanomotor design whose motion can be independently controlled in both the transversal plane and the longitudinal axis. A suitable platform for optical pulling was first developed, based on waveguide coupling between an azimuthally polarized Bessel beam and a glass cylinder coated with anti-reflective layers. Then, using this cylinder as the main chassis for the nanomotor, asymmetric plasmonic dimers were strategically placed within the dielectric cylinder to avoid strong interaction with the pulling beam and thus preserving the optical pulling effect. Therefore, a plane wave incidence produces lateral motion, driven by the lateral force from the plasmonic dimers, while incidence with a Bessel beam provides movement on the longitudinal axis via the optical pulling force on the cylinder. Our design enables the selection of motion modes: pulling, pushing, and lateral movements (left-right, forward-backward) by adjusting the polarization or switching between plane wave and Bessel beam illuminations. Furthermore, we demonstrate that the optical pulling force achieved in our system is highly robust against rotations, translations and Brownian motion, furthering the versatility and practical applicability of our device.

## 2 Conceptual overview and optical pulling platform

As showcased by authors in [30], optical pulling forces (OPFs) benefit from transversely isotropic particles (like cylinders) and beams, with the azimuthal polarization being more efficient in the compromise between pulling force and Bessel beam cone angle. These beams are characterized by an electric field

(1) $$\begin{array}{c} E\left(\rho ,\varphi ,z\right)=-iE_{0}{\text{e}}^{\text{i}k_{0}\cos\theta_{0}z}\frac{J_{0}^{\prime }\left(k_{0}\sin\theta_{0}\rho \right)}{\sin\theta_{0}}e^{im\varphi }n_{\varphi } \end{array}$$

where *E*_0_ is the amplitude, *k*_0_ is the beam wavenumber, $J_{0}^{\prime }\left(x\right)$ represents the first derivative of the Bessel function $J_{0}\left(x\right)$ and *m* is the topological charge of the beam, which will be zero hereinafter for the rest of this work. Upon interaction with these beams, dielectric cylinders with appropriate diameters (see Supplementary Information) can support fundamental waveguide modes (FWMs), helping reduce photon recoil in the structure. This effect can be enhanced with the usage of antireflection coatings (ARCs). As stated before, OPFs need collimation of the incident light, something that can happen for some values of the length of the cylindrical waveguide, owing to interference of some Fourier components of the scattered field.

**Figure 1:**
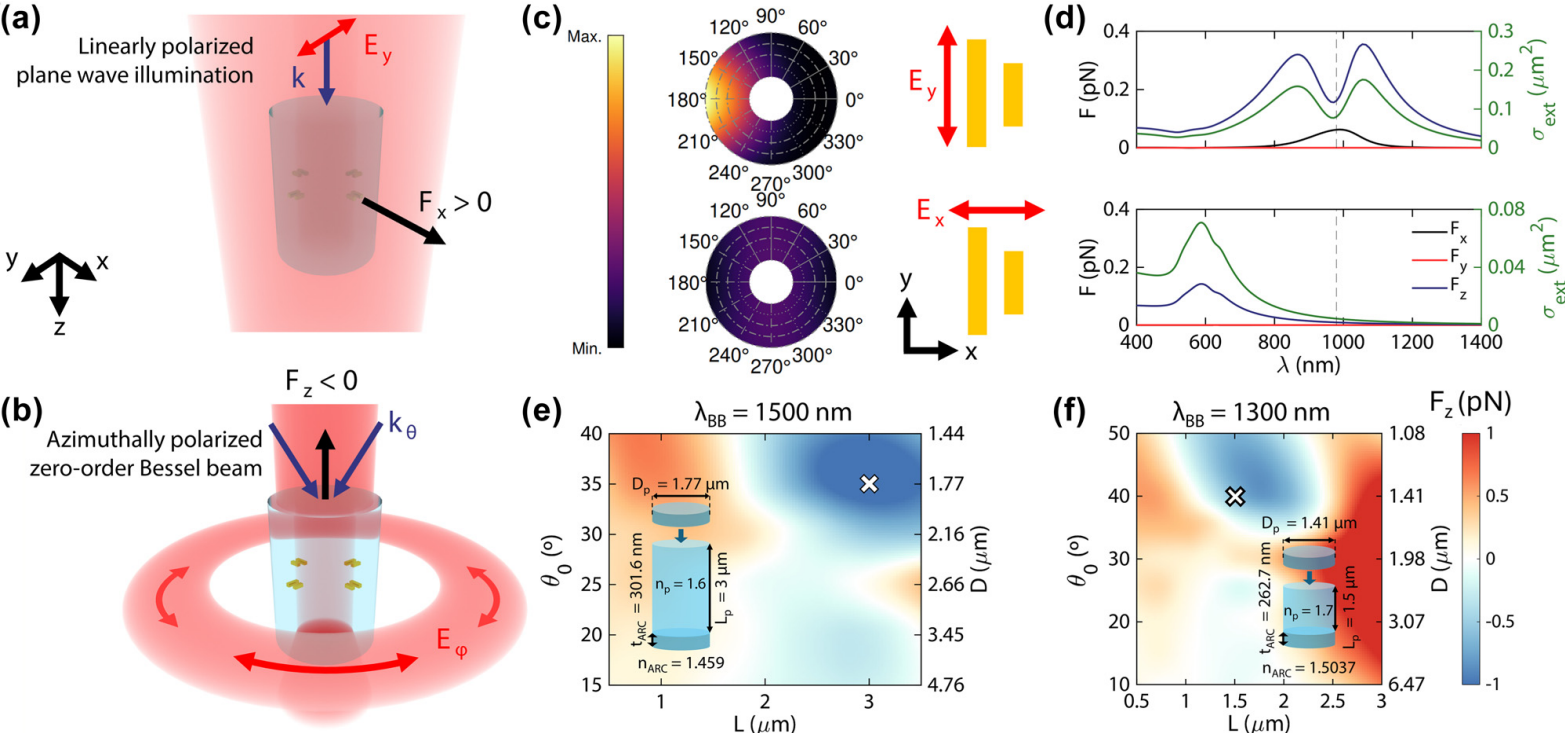
Scheme of the nanomotor’s lateral movement, provided by the plane wave illumination (a) and its pulling force mechanism with the Bessel beam (b). (c) Scattering pattern of plasmonic nanorod dimers at a wavelength of 980 nm, for both parallel (*E*_*y*_) and perpendicular polarizations (*E*_*x*_). The nanorods are characterized by lengths of 170 and 130 nm, separated by a distance of 100 nm. Their depth and width are the same, at 50 nm. (d) Optical forces and extinction cross section for the aforementioned dimers and polarizations. The illumination intensity is 0.4 mW μm^−2^. (e) Longitudinal force on a dielectric cylinder upon interaction with an azimuthally polarized Bessel beam (characterized by angle *θ*_0_) at a wavelength of 1,500 nm and a wavelength of 1,300 nm (f). The insets reflect the geometrical and material features of the two selected designs, corresponding to the white crosses in their respective maps. The different-coloured cylinders above and below correspond to the ARCs, whose index is indicated below the cylinders.

The general idea behind our design is illustrated in Figure 1(a) and (b). We exploit the efficient OPF from zero-order azimuthally polarized Bessel beams on dielectric cylinders, and we add plasmonic rod dimers to provide lateral motion. As seen in Figure 1(c), these plasmonic dimers, when illuminated with *y*-axis polarization at the superposition of their respective resonance (around 980 nm in Figure 1(c)), provide very directional lateral scattering, driving the lateral (*x*-component) force seen in Figure 1(d) [31], [32], [33]. Meanwhile, the perpendicular polarization at the same wavelength, is characterized by very low and uniform scattering, resulting in negligible optical forces, as shown in Figure 1(d).

As can be seen in Figure 1(d), the optical force exerted in these dimers has a dominant longitudinal (*z*) component, which closely follows the dimer’s extinction cross-section. This *z*-component force is also quite large for even moderate illumination intensities (0.4 mW μm^−2^), meaning that the pulling force exerted by a Bessel beam might be suppressed if strong interaction with the dimers occurs. This interaction can be avoided by employing wavelengths higher than 1,000 nm and a polarization perpendicular to the dimer’s orientation, so that no resonance is excited. To achieve a cylinder design working at such wavelengths, we explore different combinations of Bessel beams (1 mW μm^−2^ maximum intensity) with wavelengths $\lambda \in \left[1,000,1,500\right]$ nm and refractive indices for the dielectric cylinder $n_{p}\in \left[1.6,1.8\right]$ . For each of these combinations, we investigate Bessel beam angles $\theta_{0}\in \left[10{}^{\circ},50{}^{\circ}\right]$ , each of them corresponding to different cylinder diameters; and different cylinder lengths $L\in \left[0.5,3\right]$ µm. Finally, each of the cylinders is equipped with suitable ARCs (see Supplementary Information). The background refractive index is set to that of water *n*_*b*_ = 1.33.

The dynamics of a cylinder in an aqueous medium strongly depend on its geometrical aspect ratio, defined as *p* = *L*/*D*, where *L* is the cylinder’s length and *D* its diameter. To investigate such different dynamics, we select two suitable configurations characterized by different cylinder length/diameter aspect ratios, as shown in Figure 1(e) and (f). In particular, Figure 1(e) shows the longitudinal (*z*-component) force map for a 1,500 nm Bessel beam and a 1.6 index cylinder, where long cylinders are generally required to excite the FWM and generate the pulling force, with a maximum negative force of −1.2 pN, located at an angle of 35° (1.77 µm diameter) and a length of 3 µm (2.04 aspect ratio).

On the other hand, Figure 1(f) shows the force map for a 1,300 nm wavelength Bessel beam and a 1.7 index cylinder, where the maximum negative force of −0.6 pN is located at an angle of 40° (1.44 µm diameter) and a length of 1.8 µm. To ensure that we can work with a sufficiently different aspect ratio, here we select a suboptimal configuration (same diameter, length of 1.5 µm) with a lower (1.44) length/diameter aspect ratio.

## 3 Plasmonic dimer placement for optical pulling preservation and lateral motion

As stated previously, minimizing the interaction with the dimers is key to preserve the pulling force. As shown in Figure 1(d), interaction is minimized for polarizations orthogonal to the dimers, given that the wavelength is sufficiently long as to avoid the resonance of the rod’s short dimension and the intrinsic absorption of gold. However, the complex interaction of the Bessel beam with the nanomotor’s glass chassis might result in complex polarization patterns, meaning that the force exerted on the dimers can be difficult to predict.

**Figure 2:**
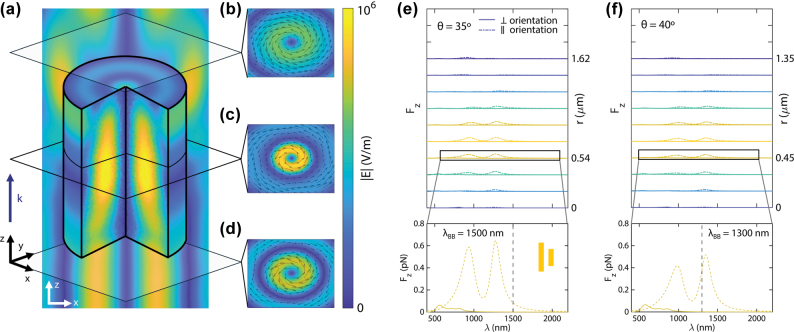
Interaction of plasmonic dimers with Bessel beams. (a) Electric field within the cylinder in Figure 1(f) (1.44 µm diameter, 1.5 µm), upon interaction with a 40°, 1,300 nm Bessel beam, with cut planes below (b), in the middle (c) and above (d) the cylinder, featuring the field polarization with black arrows. Incidence of the beam is in the positive *z* direction. (e) and (f) Wavelength dependence of longitudinal forces exerted on the asymmetric plasmonic dimers (rods 50 nm wide and deep, 170 and 130 nm long, 100 nm gap) by Bessel beams with angles *θ* = 35° (e) and *θ* = 40° (f). The dimers are located at different radial positions *r* with respect to the beam center. Solid lines represent an orientation of the rods perpendicular to the azimuthal polarization of the beams while dashed lines represent a parallel orientation. The dimers are placed in a background medium (without the pulling cylinder, hence the positive *F*_*z*_ values) with refractive indices corresponding to those of the cylinders (*n*_*p*_ = 1.6 for the 35° beam and *n*_*p*_ = 1.7 for the 40° beam).

To characterize the general properties of the incident field impinging on the dimers, we show in Figure 2(a) the electric field inside and in the vicinity of the cylinder in Figure 1(f) (1.44 µm diameter and 1.5 µm length), upon interaction with its suitable pulling Bessel beam. Cut planes below, in the middle and above the cylinder are featured in Figure 2(b)–(d), with the field’s polarization. As can be seen in Figure 2(a), the field has a similar shape to the incident beam below the cylinder, with a clear azimuthal polarization pattern in Figure 2(d). Meanwhile, in the inner part of the cylinder field gets concentrated around its center due to its higher refractive index. Due to the TE FWM excitation, the field retains its azimuthal polarization (Figure 2(c)). Finally, the field above the cylinder has a more complex shape, owing to interference with the parts of the beam that impinge laterally on the cylinder. However, a close examination in Figure 2(b) shows that the field is generally less focused due to the collimation effect and remains azimuthally polarized.

In general, the incident field on the dimers will correspond to the same profile of the incident Bessel beam, corrected by the higher refractive index of the cylinder. Thus, we can find the Lorentz’s force exerted on a single nanorod by approximating said rod as a dipole with dipolar moment ***p***. For harmonic fields, the time-averaged force $\left\langle F\right\rangle$ is [34], [35]:

(2) $$\begin{array}{c} \left\langle F\right\rangle =\frac{1}{2}Re\left[\left(p^{*}\cdot \nabla \right)E+i\omega \left(p^{*}\times B\right)\right] \end{array}$$

where *ω* is the angular frequency of light and ***B*** is its magnetic field. Using the electric field from equation (1) with *m* = 0 and letting ***p*** = ɛ_*b*_*α* ***E*** (with $\varepsilon_{b}=\varepsilon_{0}n_{b}^{2}$ ) and, from Maxwell’s equations, $B={\left(i\omega \right)}^{-1}\nabla \times E$ ; it can be shown (see Supplementary Information for the full derivation) that the total force is

(3) $$\begin{array}{c} \left\langle F\right\rangle =\frac{1}{2}Re\left[\varepsilon_{b}\alpha^{*}E_{\varphi }^{*}\left(\frac{\partial E_{\varphi }}{\partial \rho }n_{\rho }+\frac{1}{\rho }\frac{\partial \left(\rho E_{\varphi }\right)}{\partial z}n_{z}\right)\right] \end{array}$$

where *E*_*φ*_ is the azimuthal field in equation (1). This expression can be further simplified to

(4) $$\begin{array}{ll} \left\langle F\right\rangle & =\frac{1}{2}Re\left[\varepsilon_{b}\alpha^{*}E_{0}^{2}\left(\frac{k_{0}}{\sin\theta_{0}}J_{1}\left(x_{\rho }\right)J_{1}^{\prime }\left(x_{\rho }\right)n_{\rho }\right.\right.\\ & \;\left.\left.+i\frac{k_{0}\cos\theta_{0}}{\sin^{2}\theta_{0}}J_{1}^{2}\left(x_{\rho }\right)n_{z}\right)\right] \end{array}$$

where *x*_*ρ*_ = *k*_0_ sin *θ*_0_*ρ*. As can be seen, a transversal force in the radial direction arises as well as a longitudinal force. Note that the conjugate polarizability α*, at the Frölich condition of a plasmonic nanoparticle, will have a negative imaginary part [36], meaning that the longitudinal force will be positive (pushing force). Both force components can be eliminated by: (1) placing the dipole at zero field points (zeroes of the Bessel function *J*_1_(*x*_*ρ*_)), which can be impractical due to the size constraints imposed by the optical pulling force as well as fabrication issues; and (2) minimizing the polarizability, making both force components effectively zero and allowing independent control of pulling and lateral forces.

For rod-like particles, characterized by highly anisotropic polarizabilities, interaction can be suppressed by polarizing light along the shorter dimension of the rod, minimizing and shifting the resonance to lower wavelengths. Although interactions in a dimer can result in more intricate force patterns, a parallel rod dimer, as previously shown in Figure 1(d), can present a similar response to a single rod system. This means that parallel rod dimers, in contrast with other proposals, could be employed to effectively select between transversal and longitudinal motion.

Due to the non-trivial interactions between the rods in a dimer, we propose a semi-analytical coupled dipole approximation (CDA) method approach, following [37], to calculate the optical forces from such beams onto a single plasmonic dimer (see Supplementary Information for details on this method).

We select the same angles and refractive indices featured in Figure 1(e) and (f) and calculate the longitudinal optical force on a dimer formed by the same rods shown in Figure 1(d) (50 nm wide and deep, 170 and 130 nm long), and explore different radial positions within the beam, enough to cover the higher intensity regions. We also consider two different orientations, one oriented along the beam polarization and one perpendicular to it. Due to the nature of CDA simulations, the dielectric cylinder was omitted, and its refractive index (in both cases) was used as the background index for the CDA simulations.

Results are shown in Figure 2(e) (35° Bessel beam, for the 1.6 refractive index cylinder in Figure 1(e)) and Figure 2(f) (40° Bessel beam, for the 1.7 refractive index cylinder in Figure 1(f)). Both figures display a dual peak shape for the parallel orientation, following closely the shape in Figure 1(c), with *z*-component forces up to 1 pN. As expected from the single-rod behavior, the magnitude of forces correlates with the positions of higher intensities. On the other hand, the perpendicular orientation, characterized by a lower and blue-shifted polarizability, displays a flatter profile, with forces up to 0.1 pN near 500 nm, and near-zero forces for wavelengths higher than 1,000 nm. In particular, the insets of Figure 2(e) and (f) show the force profile for radial positions near the edge of their respective cylinders. These positions can be advantageous, enabling the integration of several dimers within the cylinder that can be operated independently. Despite being high-intensity sites, the exerted force is near-zero when oriented orthogonally to polarization, due to their very low and blue-shifted polarizability. Thus, optical pulling is preserved. We highlight that this result showcases the independent physical mechanisms that induce the two types of forces, with no coupling between them. Additional components for the inset cases in Figure 2(e) and (f) can be found in the Supplementary Information (Figure S5), where it can be seen that, as occurs with a single rod, the transversal forces in dimers can be avoided with a perpendicular orientation with respect to the azimuthal polarization.

**Figure 3:**
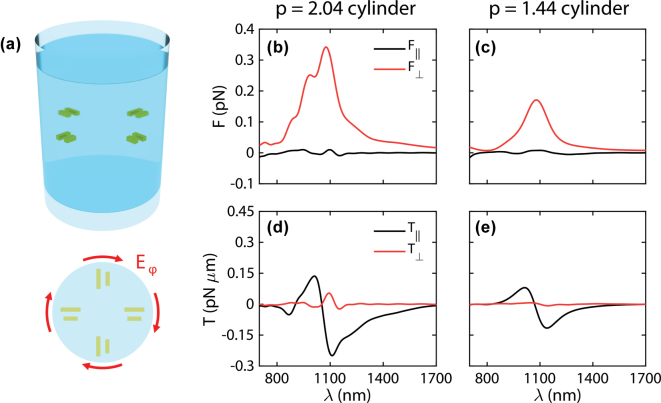
Simulation results for lateral movement. (a) Scheme of a full 3D nanomotor design, with four plasmonic dimers perpendicularly oriented to the azimuthal polarization of a pulling Bessel beam. (b) Optical transversal forces exerted by a 0.4 mW μm^−2^ plane wave on the nanomotors based on cylinders with aspect ratios 2.04 and 1.44 (c). (d) Optical transversal torques exerted by a plane wave on the same nanomotors. (e). The cylinder with aspect ratio 2.04 contains three layers of plasmonic dimers, in contrast with the 1.44 aspect ratio cylinder, which only includes one. The background refractive index is *n*_*b*_ = 1.33.

Due to the axial symmetry of this design, we can position several dimers within the cylinder, making sure that all of them are oriented perpendicular to the Bessel beam polarization. An illustration of this configuration is shown in Figure 3(a). In this case, the use of 4 dimers allows independent control of movement in the *x* and *y* directions upon linearly-polarized plane wave illumination. This is supported by Figure 3(b)–(e), where the optical forces and torques on both cylinders are shown. As expected, the component of force perpendicular to polarization dominates, with a clear peak around 1,100 nm. On the other hand, forces on the polarization axis are small, meaning that movement between the two directions can be independently controlled. For both designs, forces are in the 0.1 pN range, sufficient to overcome Brownian motion and obtain a steady movement speed [12].

Looking at the torques, the parallel component dominates, with an oscillating shape around the same wavelength. While the peaks for the perpendicular force component fall within positive torque values (meaning that the cylinder could tumble with time), this effect can be suppressed by using slightly different wavelengths, where the torque can be suppressed.

The longitudinal components of both force and torque (*F*_*z*_ and *T*_*z*_) are provided in the Supplementary Information (Figure S6). The longitudinal force is much larger than either *F*_‖_or *F*_⊥_, pointing to strong forward movement when using plane wave illumination. As shown in the Supplementary Information, control of the pushing movement can be done independently of lateral movements if out-of-resonance wavelengths are employed. On the other hand, the longitudinal torque *T*_*z*_ is around 10 times weaker than the *T*_‖_ component in Figure 3, meaning that for such illumination *T*_‖_ is the only relevant component.

The scheme described so far only provides translational motion, but a similar strategy can be employed to implement rotational motion. As shown in the Supplementary Information (Figure S8), silver nanorods can be inserted between the dimers. Illumination with circularly polarized light allows transfer of spin angular momentum into the nanorods [38], [39], [40], [41], permitting efficient clockwise and anticlockwise rotations depending on the incident helicity. Furthermore, by placing the rods perpendicular to the azimuthal polarization, the optical pulling force is still preserved.

## 4 Optical pulling stability in diffusion simulations

The longitudinal force maps, shown in Figure 1(e) and (f), correspond to cylinders fully aligned with the incident beam. However, when nanomotors are immersed in a fluid such as water, they can undergo Brownian motion, meaning that the pulling must be preserved upon small displacements and rotations from the beam center. Furthermore, as shown in Figure 3, interaction with light can induce transversal forces and torques, further prompting the need for stable pulling forces for a realistic application of these nanomotors. In particular, and contrary to optical tweezers, the incident Bessel beams have a ring-shaped maximum intensity, leading to the possibility of gradient optical forces driving the nanomotor away from the beam center [42], [43].

To elucidate the impact of these rotations, we performed FDTD simulations considering displacements from the center along the *x* axis. As the cylinder moves along the *x* axis and away from the center, the incident azimuthal polarization becomes similar to a linear *y*-polarization. Inspired by Figure 3, both the *x*-component force and the *y*-component torque will become important and dominate against the other transversal components. Therefore, rotations around the *y* axis are also considered in simulations.

**Figure 4:**
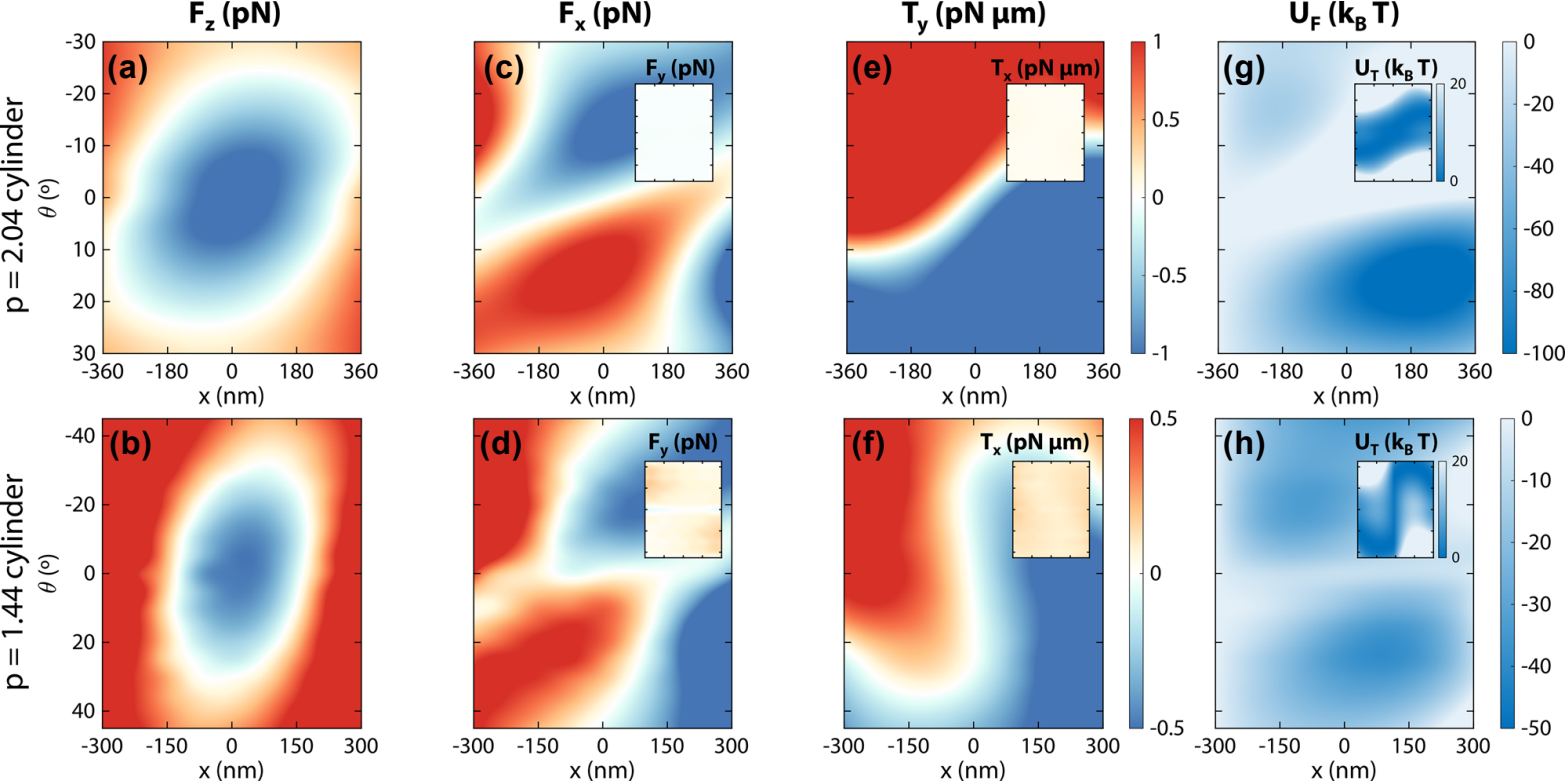
Position and angular dependence of the optical pulling scheme. (a) and (b) Longitudinal force exerted on the *p* = 2.04 and 1.44 nanomotors by their corresponding Bessel beams at different *x* positions and tumbling angles *θ*. (c) and (d) Dominant transversal optical forces (*x*-component), with the negligible *y*-component optical forces shown in the respective insets. (e) and (f) Dominant transversal optical torques (*y*-component), with the negligible *x*-component optical forces shown in the respective insets. (g) and (h) Potential maps associated with the transversal force (*U*_*F*_), with the potential associated to the torques (*U*_*T*_) in the insets. Simulations for this figure include the full 3D-nanomotor system as in Figure 3 (dielectric cylinder plus plasmonic dimers) in a background medium with refractive index *n*_*b*_ = 1.33.

The stability of the optical pulling effect provided by the Bessel beams is showcased in Figure 4(a) and (b). While a maximum negative force is found at the beam center, pulling is preserved to rotations up to 20° in both cylinders, and for up to 150–200 nm displacements. The expected dominant transversal components of force and torque are shown in Figure 4(c) and (d) (transversal force) and Figure 4(e) and (f) (transversal torque), with insets depicting the non-dominant components. As shown in the insets, the non-dominant components are homogeneous and their contribution can generally be neglected against the dominant components, especially in the case of the longer nanomotor. In contrast, the dominant components of both force and torque resemble those observed in optical tweezers in the ray optics regime [44], leading to equilibrium positions and orientations. The *x*-component force reaches zero around the center of the beam, with the position of this zero slightly shifting to the left or right depending on the nanomotor angular orientation. Similarly, the *y*-component torque reaches zero for most angles at the beam center, and this zero is shifted to angles up to 10° (*p* = 2.04 nanomotor, 40° for the *p* = 1.44 one) outside the beam center. The non-dominant longitudinal torque *T*_*z*_ can be consulted in the Supplementary Information. Once again, the near-zero lateral forces and torques at the beam’s positional and angular center highlight the negligible coupling between the optical pulling force and the lateral forces observed in Figure 3.

These zero positions and orientations point towards equilibrium points of the nanomotor when illuminated with the Bessel beams. For a further understanding of these restoring forces and torques, we compute potentials from both the dominant transverse forces and torques by integration $U_{F}\left(x,\theta \right)=-\int_{x_{\min}}^{x_{\max}}F_{x}\left(x,\theta \right)dx$ and $U_{T}\left(x,\theta \right)=-\int_{\theta_{\min}}^{\theta_{\max}}T_{y}\left(x,\theta \right)\sin\left|\theta \right|d\theta$ . The force-related potential $U_{F}\left(x,\theta \right)$ is represented in Figure 4(g) and (h), with the torque-related potential $U_{T}\left(x,\theta \right)$ in the respective insets. The *U*_*F*_ potentials are characterized by two wide potential wells, with centers located around ±180 nm and ±20° (longer design), and ±100 nm and ±30° (shorter design). On the other hand, the *U*_*T*_ potentials follow the zero-line shape of the torque more closely, resulting in potential wells at positive angles for negative *x* positions and negative angles for positive *x* positions. We must highlight that both potentials have values well over 10 *k*_*B*_*T* meaning that Brownian motion should have little influence on the overall system dynamics.

Ultimately, the closeness of the integration domain to the beam center and the difficulty to apply appropriate boundary conditions to the angular integration in *U*_*T*_, disallows adding the two potentials together for a quantitative total potential, meaning that we must make a qualitative interpretation of their shapes.

In principle, the *U*_*T*_ wells, at opposite positions from the ones in *U*_*F*_, should balance a total potential, allowing the nanomotor to travel across all the central region. To address rigorously the non-trivial dynamics in this system, we set up hydrodynamic diffusion simulations (details can be found in the Methods section as well as in the Supplementary Information). We assume an overdamped low-Reynolds-number regime, with external forces given by the dominant components of force and torque from Figure 4. To evaluate the stability of the system, we target a long illumination time of 1 s and run 10,000 simulations per case in order to obtain significative statistical information.

**Figure 5:**
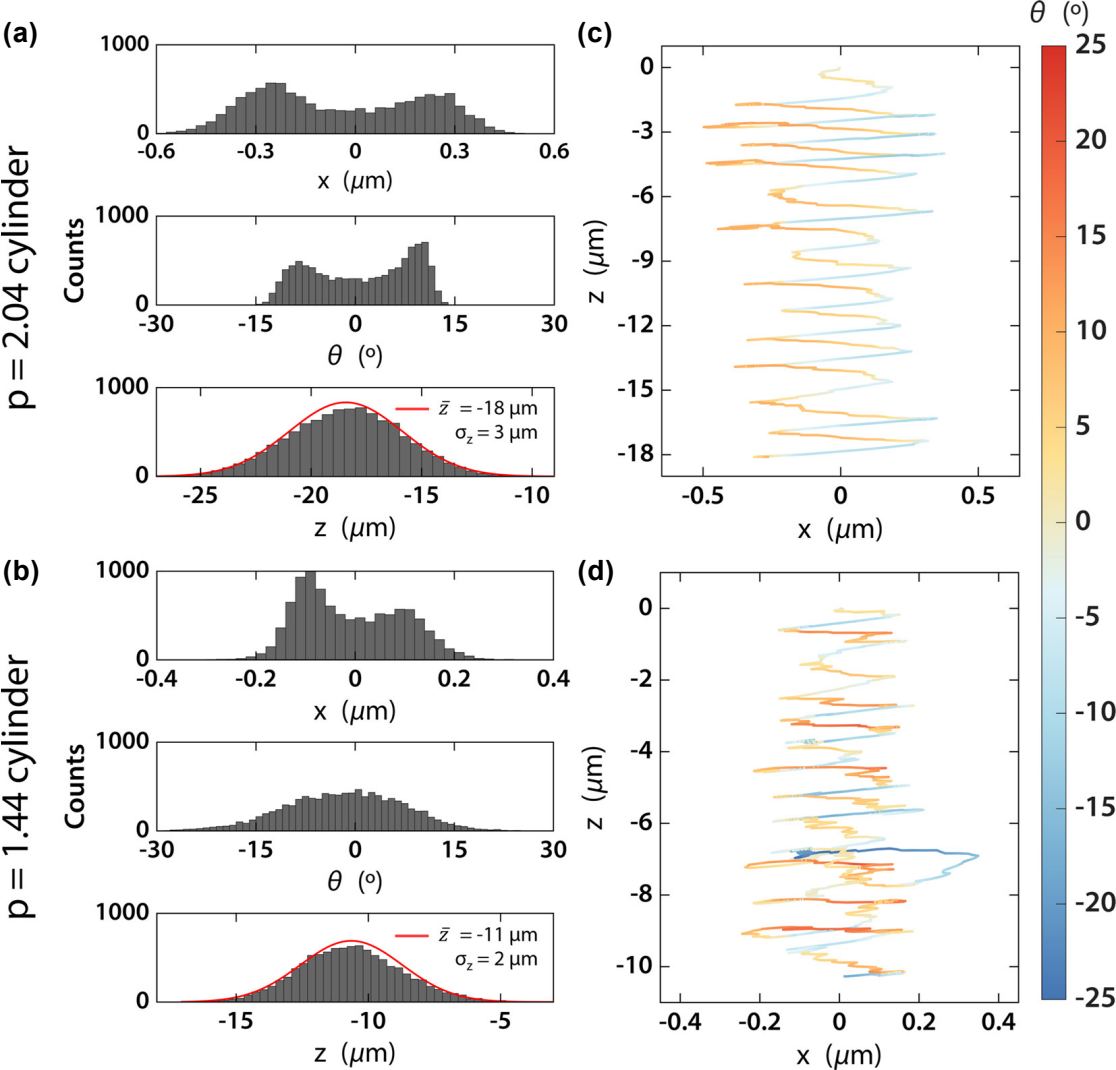
Results of hydrodynamic simulations. (a) and (b) Histograms of the final positions, orientations and longitudinal displacement for the *p* = 2.04 (a) and 1.44 (b) aspect ratio nanomotors after 1 s illumination. Gaussian fittings are provided for longitudinal positions. (c) and (d) Typical trajectories of the nanomotors, starting at $\left(x,\theta ,z\right)=\left(0,0,0\right)$ and travelling downwards while oscillating in both *x* and *θ*.

The overall stability of the nanomotors is demonstrated by the results shown in Figure 5. As can be seen in the upper panels of Figure 5(a) and (b), nanomotors are distributed around two different positions (one positive, one negative), which is compatible with the two different potential wells in both Figure 4(g) and (h). The longer nanomotor design also follows a similar shape in its angular distribution. However, the shorter design displays a less bi-morph distribution, with a 0° maximum. This can be explained by the similar *U*_*T*_ wells of both designs. As *U*_*F*_ wells are much deeper in the first design, the contribution from *U*_*T*_ does not allow easy passage between the two potential wells.

Meanwhile, the much lower and homogeneous *U*_*F*_ wells for the shorter design point to a joint total potential near the beam center and low angles. This bigger contribution from *U*_*T*_ in the shorter nanomotor also creates a more homogeneous angular profile in the total potential, which is reflected by the wider angular distribution in Figure 5(b).

This is further confirmed by the typical trajectory of nanomotors, displayed in Figure 5(c) and (d) and characterized by an oscillation between the two different *x* positions, together with small angular oscillations. This results in the nanomotor being in the pulling region in Figure 4(a) and (b) for most of the illumination time. This, as shown in the lower panels of Figure 5(a) and (b), results in large pulling distances, characterized by Gaussian distributions around 18 and 11 µm, with standard deviations of 3 and 2 µm, respectively. Although the range of both distributions is relatively large (over 10 µm), the standard deviations are comparatively small, leading to moderately narrow distributions. It must be noted that the average values correspond to several times the lengths of the nanomotors, meaning that the speed of nanomotors pulling with moderate intensities is relatively fast. Note that gravitational effects, which could suppose a force contribution close to 0.1 pN (nanomotor masses can be estimated around 10 pg), have been neglected in this analysis. Assuming optical pulling is acting against it, gravitational drag could significantly slow this movement. However, its low magnitude against the optical pulling force means that it can be compensated using a slightly higher illumination intensity.

## 5 Conclusions

In this work, we have revealed how optical pulling can be integrated synergically into transverse light-driven nanomotors, unlocking a new and important degree of freedom to optical manipulation. Optical pulling can be attained employing azimuthally polarized Bessel beam illumination on carefully designed dielectric cylinders, where a FWM is excited and interference at its end produces a collimation of the incident beam. This scheme allows for an efficient generation optical pulling forces, where high-angle Bessel beams are not required and microscale pulling becomes practical. To combine this approach with transverse movements, plasmonic rod dimers were embedded perpendicular to the azimuthal polarization to allow preservation of the optical pulling effect due to the weak plasmonic interaction. Candidate materials for dielectric cylinders that generate optical pulling force include SU-8, Al_2_O_3_, and GaN, which are transparent in the visible and near-infrared regions and can be fabricated with high aspect ratios [45], [46], [47]. Additionally, the plasmonic rod dimers can be precisely embedded into the cylinder of these materials through double-exposure electron beam lithography [11], [12], [13], [14]. Experimental realization of Bessel beams with azimuthal polarizations has been done with appropriate metasurfaces [48].

Then, upon illumination with linearly polarized plane waves, the asymmetric scattering from the metallic dimers results in two independent lateral motions, which can be selected with the incident polarization. Pushing motion can also be obtained as a byproduct of the plane wave illumination for lateral movement and can also be independently controlled by shifting the incident wavelength. As demonstrated in the Supplementary Information, complementary structures can be implemented into the design to efficiently provide controlled rotations [33], [38], [39], [40], [41], which can be independently controlled from other degrees of freedom upon tuning their plasmonic resonances to a different wavelength than the one for translation movement. This can be accomplished using different sizes or materials [36].

Finally, we have demonstrated how this approach is robust enough against rotations, displacements and misalignments, which can be caused by either Brownian motion, the complex Bessel beam profile, or a combination of both. The restoring nature of the transversal forces and torques exerted by the beam produces an intricate potential profile, where the nanomotors oscillate around the beam center while maintaining a straight orientation over long times. This stability, together with the low intensities required for manipulation, greatly enhances the applicability of our approach. We note that other nanomotor designs are characterized by lower height/lateral size aspect ratios (for example, *p* < 0.1 in [13]). However, these aspect ratios do not consider the strict conditions imposed by optical pulling forces. Nonetheless, this could also be reached by our design, if the wavelength of the Bessel beam is drastically lowered. For that, the plasmonic dimers should be tuned to UV wavelengths, for which the use of materials different than gold would be required [49], [50].

## 6 Methods

### 6.1 Numerical electrodynamic simulations

All numerical electromagnetic calculations in this work, excepting those specified with the Couple Dipole Approximation approach in Figure 2 (a detailed discussion can be found in the Supplementary Information), were performed by 3D full-wave electrodynamic simulation software Lumerical FDTD, providing an accurate solution of the Maxwell equations. The non-diffracting Bessel beams were implemented into FDTD using a combination of 60 different total field/scattered field (TFSF) sources, following [51] (more details in the Supplementary Information). The angular dispersion for broadband sources in Lumerical FDTD meant that simulations had to be single-frequency to provide accurate results. This motivated us to use the CDA approach for broadband simulations when considering the plasmonic dimer. Calculation of optical forces ***F*** and torques ***T*** was done following the Maxwell stress tensor formalism [44]:

(5) $$\begin{array}{c} F={∯}_{\Sigma }\bar{T_{M}}\cdot nd\Sigma \end{array}$$

(6) $$\begin{array}{c} T=-{∯}_{\Sigma }\left(\bar{T_{M}}\times r\right)\cdot nd\Sigma \end{array}$$

where ***r*** is the position vector, ***n*** is the outward normal unit vector and the integration is performed over a surface Σ enclosing the nanomotor. $\bar{T_{M}}$ is the time-averaged Maxwell stress tensor:

(7) $$\begin{array}{ll} \bar{T_{M}} & =\frac{1}{2}Re\left[\varepsilon_{r}\varepsilon_{0}E⊗E^{*}+\mu_{0}H⊗H^{*}\right.\\ & \;\left.-\frac{1}{2}\left(\varepsilon_{r}\varepsilon_{0}E⊗E^{*}+\mu_{0}H⊗H^{*}\right)\bar{I}\right] \end{array}$$

where *ɛ*_*r*_ is the relative permittivity, ⊗ denotes the dyadic product, ∗ the complex conjugate and $\bar{I}$ is the identity dyadic.

### 6.2 Hydrodynamic simulations

Hydrodynamic simulations were done following the formalism for diffusion of non-spherical particles in [44]. Due to the axial symmetry and the prevalence of *x*-component forces and *y*-component torques against other components, diffusion along the *x* and *z* axes, and increments in the *β* angle (tumbling along the *x* axis) were considered. Assuming a low-Reynolds-number regime, with external forces *F*_*x*_, *F*_*z*_ and torques *T*_*y*_, the movement of the particle after a time step Δ*t* will be given by the overdamped Langevin equation

(8) $$\begin{array}{c} \left[\begin{array}{c} \Delta x_{p}\\ \Delta z_{p}\\ \Delta \beta_{p} \end{array}\right]=\frac{\bar{D}}{k_{B}T}\Delta t\left[\begin{array}{c} F_{x,p}\\ F_{z,p}\\ T_{y,p} \end{array}\right]+\sqrt{2\Delta t}\left[\begin{array}{c} w_{x}\\ w_{z}\\ w_{\beta } \end{array}\right] \end{array}$$

where the *p* subindex denotes the particle’s frame of reference. *k*_*B*_ is the Boltzmann constant and *T* is the environment temperature. $\bar{D}$ is the particle’s characteristic diffusion tensor, which for cylindrical particles in the considered dimensions has the form

(9) $$\begin{array}{c} \bar{D}=\left[\begin{array}{ccc} D_{t} & 0 & 0\\ 0 & D_{t} & 0\\ 0 & 0 & D_{r}^{⊥} \end{array}\right] \end{array}$$

Following [52], each of the components of this diffusion tensor will be strongly dependent on the cylinder aspect ratio *p*, and can be extracted using interpolating equations. Finally, *w*_*x*_, *w*_*z*_ and *w*_*β*_, accounting for Brownian motion; correspond to a set of random numbers extracted from a multivariate normal distribution with mean zero and covariance $\bar{D}$ .

For each time step (Δ*t* = 0.001 s), external forces and torques are extracted from FDTD results based on the particle’s position and orientation, and converted to the particle’s frame of reference. Particle position and orientation are updated based on equation (8), and then the particle’s frame of reference is updated accordingly.

We note that the overdamped approximation in equation (8) must be justified by the ratio of the characteristic time of an optical trap *τ*_*OT*_ = *γ*/*κ* and the momentum relaxation time *τ*_*m*_ = *m*_*NM*_/*γ*, where $\gamma =\frac{k_{B}T}{D_{t}}$ is the friction coefficient, *κ* is the trap stiffness and *m*_*NM*_ is the nanomotor mass [44]. As can be derived from the Supplementary Information, *γ* ∼ 10^−8^ kg s^−1^; while the nanomotor masses can be estimated to *m*_*NM*_ ∼ 10^−14^ kg by approximating them to glass cylinders with density *ρ* = 2,500 kg m^−3^. Estimation of the trap stiffness is not trivial, given the complex dependence of forces and torques on position and angle. However, the force magnitude in the pN range, compared to 10^−7^ m displacements, suggests stiffnesses of up to around 10^−5^ N m^−1^. These estimated quantities yield approximately characteristic times *τ*_*OT*_ ∼ 10^−3^ s, much higher than the momentum relaxation time *τ*_*m*_ ∼ 10^−6^ s, thus justifying the overdamped treatment and the choice of the time step.

## Supplementary Material

Supplementary Material Details
