# Supplementary material for: 3-dimensional plasmonic nanomotors enabled by independent integration of optical pulling and lateral forces
Source: Nanophotonics. 2025 Sep 22;14(20):3339–50. doi: 10.1515/nanoph-2025-0374 (PMC12588568; doi:10.1515/nanoph-2025-0374)
Supplement: Supplementary file 1 — Supplementary Material Details [file j_nanoph-2025-0374_suppl_001.pdf]

Supplementary Information

Guillermo Serrera, Yoshito Y. Tanaka and Pablo Albella

3-dimensional plasmonic nanomotors driven by Optical Pulling Forces

S1. Excitation of the FWM and additional suitable designs

We follow the basic theory of waveguide coupling [1] and apply it to the dielectric cylinder as done in [2]. For such a cylinder, both TM and TE waveguide modes. Particularly, an l −order TE mode in a waveguide with radius a will be described by a tangential electric field

$$E_\varphi(\rho,\varphi,z) = \left[-\frac{ik_z a^2 l}{u\rho^2} A J_l\left(\frac{u\rho}{a}\right) + \frac{i\omega\mu a}{u} B J'_l\left(\frac{u\rho}{a}\right)\right]\cos(l\varphi)\, e^{ik_z z} \qquad (S1)$$

where k_z is the propagation constant, ω is the angular frequency of the wave and $u^2 = \left(k_p - k_z^2\right)a^2$and A and B are constants. For $l = 0$, this transforms into

$$E_\varphi(\rho,\varphi,z) = \frac{i\omega\mu a}{u} B J'_0\left(\frac{u\rho}{a}\right) e^{ik_z z} \qquad (S2)$$

which has a similar spatial profile as the azimuthally polarized Bessel beam (itself a TE wave, characterized by a purely transversal E_φ) in **equation 1** in the main text. This similar polarization and spatial profile mean that the coupling efficiency η, expressed through the overlap integral at the waveguide cross-sectional surface Σ [3]

$$\eta \propto \left|\iint_\Sigma \boldsymbol{E}^*_{\boldsymbol{BB}}(\rho,\varphi)\cdot \boldsymbol{E}_{\boldsymbol{TE}}(\rho,\varphi) d\Sigma\right|^2 \qquad (S3)$$

will be maximized. This maximum coupling, in turn, suppresses photon recoil, favoring the optical pulling effect. A similar phenomenon occurs when coupling a radially polarized (TM) Bessel beam with a TM-supporting waveguide, hence the viability of radially polarized beams for optical pulling with small-angle Bessel beams.

Thus, cylinders must be able to support the TE mode in order to maximize the pulling effect. The cutoff condition for such excitation is given by the solution of the equation

$$\frac{J_1(u)}{J_0(u)} = -\frac{u}{w}\frac{K_1(w)}{K_0(w)} \qquad (S4)$$

where $w = \left(k_z^2 - k_b\right)a^2$ and $K_l(x)$ is the l −order modified Bessel function of the second kind. For an incident Bessel beam with incidence angle θ_0 , $k_z = k_p \cos\left(\theta_p\right)$, and for small θ_p, an approximate solution is given by

$$D(\theta_0) = \frac{2z_1}{k_b\theta_0} \qquad (S5)$$

where z_1 is the first zero of the Bessel function $J_1(u)$. However, greater incidence angles require a full numerical solution of **equation S4**. This is evidenced in **Figure S1**, where the solution of **equation S4** for a wavelength of 1500 nm and indices $n_b = 1.33$ and $n_p = 1.6$ is shown, together with the approximation in **equation S5**. While the overall shapes of the solutions are similar, and values for small angles have little discrepancy, this difference becomes important once high angles are reached. The inset zooms in both solutions for angles between 30 and 40º, where the difference between solutions is approximately 0.5 μm, around 30% in relative error.

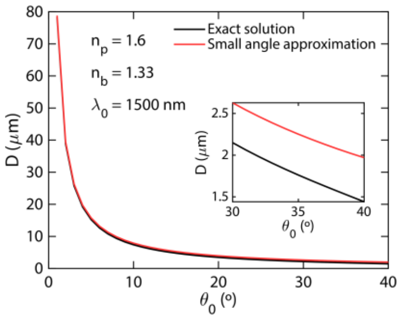

Fig. S1. Comparison of the solution of **equation S4** and **equation S5** for a wavelength of 1500 nm and indices combination $n_b = 1.33$ and $n_p = 1.6$. The inset zooms in the $\theta_0 \in [30,40]$º range, where differences can be appreciated better. The 1.77 µm diameter used for the cylinder in Figure 1e in the main text can be extracted from the black line at $\theta_0 = 35$º.

Not all beams can be coupled to the mode. For each refractive index combination, there exists a critical angle $\theta_{crit} = \text{asin}\left[\sqrt{\left(\frac{n_p}{n_b}\right)^2 - 1}\right]$ over which the FWM cannot be excited, putting an upper bound to the exploration of different cone angles [4]. For example, for $n_p = 1.6$ and $n_b = 1.33$, $\theta_{crit} \approx 42$º, and for $n_p = 1.5$; it drops to $\theta_{crit} \approx 31$º. On the other hand, angles over 45º quickly suppress the range of the Bessel beam. Therefore, our search has limited to $n_p \in [1.6, 1.8]$ and angles $\theta_0 \in [10, 50]$º, although angles over 40º have been discarded in the $n_p = 1.6$ case due to being close to the critical angle.

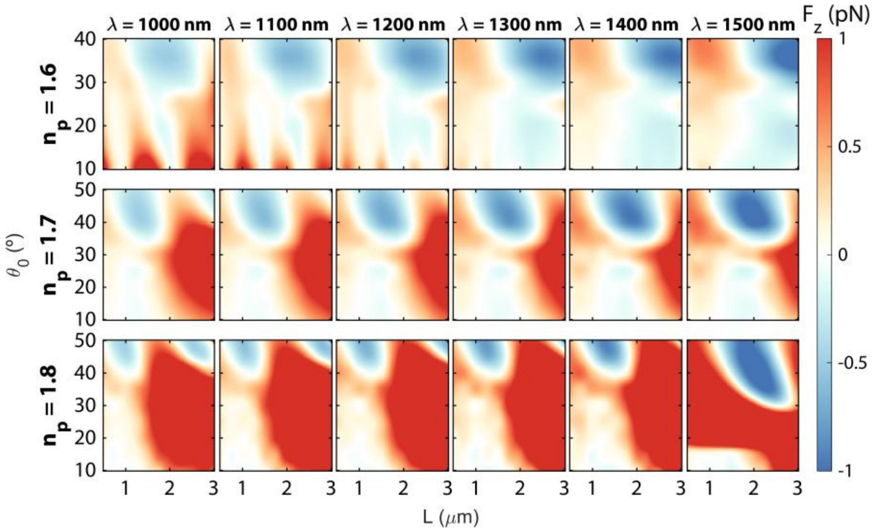

Fig. S2. Phase maps of longitudinal optical force for the whole explored casuistry. The background index is $n_b = 1.33$ for all cases.

Figure S2 shows the phase maps for the whole explored casuistry. In general, longer wavelengths and greater refractive index correspond to stronger pulling forces at their minima. High refractive index contrasts also help to bring the minima to shorter disks. For the shorter wavelengths and higher refractive indices the oscillation of the force at high angles, between pulling and pushing, becomes more visible. This is due to interference, and the period of the oscillation follows $\Delta = \frac{\lambda}{n_p - n_b}$ [5]. Also, pulling force at lower angles becomes more viable at lesser refractive index contrasts, as reflections are suppressed more efficiently by the ARCs.

S2. Antireflection Coatings design

To improve the pulling efficiency of our nanomotors, each of the cylinders analyzed in this work have been equipped with antireflection coatings (ARCs) at both ends. This strategy has been successfully employed in other works [6], [7]. The refractive index of an ARC is given by

$$n_{ARC} = \sqrt{n_p n_b} \tag{S6}$$

while its thickness is given by

$$d(\theta_0) = \frac{\lambda \cos\theta_{ARC}}{4(n_{ARC} - n_b \sin\theta_0 \sin\theta_{ARC})} \tag{S7}$$

where the propagation angle within the ARC, θ_{ARC}, is given by Snell's law:

$$\theta_{ARC} = \operatorname{asin}\left(\frac{n_b \sin\theta_0}{n_{ARC}}\right) \tag{S8}$$

Thus, each of the refractive combinations and wavelengths (each panel in Figure S2), requires ARCs with different refractive indices and/or thicknesses.

S3. Implementation of Bessel beams in FDTD

Due to the high cone angles considered in this work, a non-paraxial implementation of Bessel beams in FDTD was needed. The approach described in [8] was followed. Any arbitrary Bessel beam can be understood, in the Angular Spectrum Representation (ASR), as a superposition of plane waves with wavevectors lying on a conical surface (defined by the cone angle θ_0). In a spherical coordinate system with position vector $\boldsymbol{r}$, at the focal point of a lens, the field of an m −order Bessel beam propagating along *z* is

$$\boldsymbol{E}(\boldsymbol{r}) = \frac{ikf e^{-ikf}}{2\pi} \int_{\theta}^{\theta_{max}} \int_{0}^{2\pi} \boldsymbol{E_{PW}}(\theta,\varphi) e^{i\boldsymbol{k}\cdot\boldsymbol{r}} \sin\theta \, d\theta d\varphi \tag{S9}$$

where f is the focal length of the lens, and the wavevector $\boldsymbol{k} = (k\sin\theta\cos\varphi, k\sin\theta\sin\varphi, k\cos\theta)$. The angular spectrum function $\boldsymbol{E_{PW}}$ is given by [9]

$$\boldsymbol{E_{PW}}(\theta,\varphi) = E_{PW0}(\theta_0,\varphi) e^{im\varphi} \frac{\delta(\theta-\theta_0)}{\sin\theta} \boldsymbol{Q}(\theta_0,\varphi) \tag{S10}$$

where E_{PW0} is the amplitude of each plane wave that contributes to the beam, $\delta(\theta-\theta_0)$ is the Dirac delta distribution, limiting contributions to the cone surface with angle θ_0; and the $\boldsymbol{Q}(\theta_0,\varphi)$ vector describes polarization as [10]

$$\boldsymbol{Q}(\theta_0,\varphi) = \begin{bmatrix} p_X(\cos\theta_0\cos^2\varphi + \sin^2\varphi) - p_y(1-\cos\theta_0)\sin\varphi\cos\varphi \\ -p_X(1-\cos\theta_0)\sin\varphi\cos\varphi + p_y(\cos\theta_0\sin^2\varphi + \cos^2\varphi) \\ -p_x\sin\theta_0\cos\varphi - p_y\sin\theta_0\sin\varphi \end{bmatrix} \tag{S11}$$

and depends on the pair of parameters (p_x, p_y). An azimuthal polarization is given by $(p_x, p_y) = (-\sin\varphi, \cos\varphi)$. Then, the implementation of Bessel beams consists in introducing a combination of plane waves whose amplitude, direction and polarization matches the angular spectrum function $\boldsymbol{E_{PW}}$. The finiteness of the number of plane waves means that the integral in equation S9 will be discretized as

$$\boldsymbol{E}(\boldsymbol{r}) = \sum_{n=0}^{N} C_n E_{PW0} \boldsymbol{Q}(\theta_0,\varphi_n) e^{im\varphi} e^{i\boldsymbol{k}\cdot\boldsymbol{r}} \tag{S12}$$

where φ_n corresponds to the azimuthal angle integration points, weighted by coefficients C_n. They can be determined as

$$
\varphi_n = \frac{2\pi n}{N}, \qquad n = 0,1 \dots N
$$
$$
C_n = \begin{cases} \dfrac{\pi}{N}, & n = 0, N \\ \dfrac{2\pi}{N}, & n = 1,2 \dots N-1 \end{cases} \tag{S13}
$$

where the half-valued C_n coefficients at $n = 0, N$ compensate the degeneracy at that point. To introduce these plane waves without periodic boundary conditions in FDTD, the Total Field/Scattered Field (TFSF) within the Lumerical FDTD software approach is followed. As shown in [8], a combination of 60 plane wave sources is enough to closely match the analytical profile of the beam. Typically, injection angles in broadband simulations change as a function of frequency, with only the center frequency matching the nominal injection angle. While this dispersion can be negligible for small angles, steep incidences cause notable differences. This effect can be corrected using the Broadband Fixed Angle Source Technique (BFAST) or using frequency dependent profiles [11], these approaches are not implemented for TSFS sources, meaning that simulations had to be limited to a single frequency, matching the nominal angle.

Figure S3 displays the excellent match between the analytical Bessel beam from equation 1 and the TFSF approach in Lumerical FDTD for a zero-order azimuthally polarized Bessel beam with $\theta_0 = 55º$ at a wavelength of 532 nm.

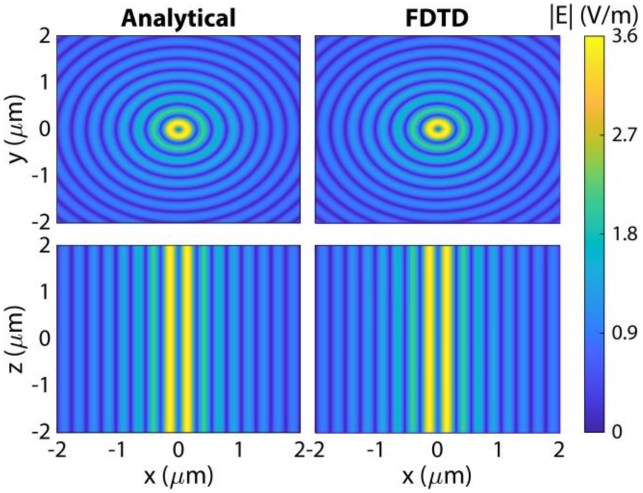

Fig. S3. Comparison of analytical and FDTD-generated Bessel beams, at XY and XZ planes. The beam is zero-order with azimuthal polarization, and is characterized by an angle of 55º, a wavelength of 532 nm, and the plane wave amplitude is set to $E_{PW0} = 1$ V/m. The surrounding medium has an index $n_b = 1.33$.

S4. Derivation of the force exerted by a zero-order azimuthally polarized Bessel beam on a plasmonic particle

We start by considering the Lorentz force on a point dipole $\boldsymbol{p}$, given by [12], [13]:

$$
\langle \boldsymbol{F} \rangle = \frac{1}{2} \Re\mathrm{e}[(\boldsymbol{p}^* \cdot \nabla)\boldsymbol{E} + i\omega(\boldsymbol{p}^* \times \boldsymbol{B})] \tag{S14}
$$

where $\boldsymbol{E}(\rho, \varphi, z) = -iE_0 e^{ik_0 \cos\theta_0 z} \frac{J_0'(k_0 \sin\theta_0 \rho)}{\sin\theta_0} \boldsymbol{n}_{\boldsymbol{\varphi}} = E_\varphi \boldsymbol{n}_{\boldsymbol{\varphi}}$, $\boldsymbol{p} = \varepsilon_\mathrm{b} \alpha \boldsymbol{E}$ (where $\varepsilon_\mathrm{b} = \varepsilon_0 n_b^2$) and $\boldsymbol{B} = \frac{1}{i\omega} \nabla \times \boldsymbol{E}$. We can substitute and obtain:

$$
\langle \boldsymbol{F} \rangle = \frac{1}{2} \Re\mathrm{e}[\varepsilon_\mathrm{b} \alpha^* (\boldsymbol{E}^* \cdot \nabla)\boldsymbol{E} + \alpha^* (\boldsymbol{E}^* \times (\nabla \times \boldsymbol{E}))] \tag{S15}
$$

Because the field only has the azimuthal component, the dot product in the first term simplifies to

$$(\boldsymbol{E}^* \cdot \nabla)\boldsymbol{E} = E_\varphi^* \frac{1}{\rho}\frac{\partial E_\varphi \boldsymbol{n_\varphi}}{\partial \varphi} = E_\varphi^* \frac{1}{\rho}\left(\frac{\partial E_\varphi}{\partial \varphi}\boldsymbol{n_\varphi} - E_\varphi \boldsymbol{n_\rho}\right) = -\frac{|E_\varphi|^2}{\rho}\boldsymbol{n_\rho} \quad (S16)$$

where in the last step the fact that the field has cylindrical symmetry $\left(\frac{\partial E_\varphi}{\partial \varphi} = 0\right)$ has been exploited. For the other term in the equation, we can first compute $\nabla \times \boldsymbol{E}$:

$$\nabla \times \boldsymbol{E} = \left(-\frac{1}{\rho}\frac{\partial(\rho E_\varphi)}{\partial z}, 0, \frac{1}{\rho}\frac{\partial(\rho E_\varphi)}{\partial \rho}\right) \quad (S17)$$

With this, the product $\boldsymbol{E}^* \times (\nabla \times \boldsymbol{E})$ yields:

$$\boldsymbol{E}^* \times (\nabla \times \boldsymbol{E}) = \begin{vmatrix} \boldsymbol{n_\rho} & \boldsymbol{n_\varphi} & \boldsymbol{n_z} \\ 0 & E_\varphi^* & 0 \\ -\frac{1}{\rho}\frac{\partial(\rho E_\varphi)}{\partial z} & 0 & \frac{1}{\rho}\frac{\partial(\rho E_\varphi)}{\partial \rho} \end{vmatrix} = E_\varphi^* \frac{1}{\rho}\frac{\partial(\rho E_\varphi)}{\partial \rho}\boldsymbol{n_\rho} + E_\varphi^* \frac{1}{\rho}\frac{\partial(\rho E_\varphi)}{\partial z}\boldsymbol{n_z} \quad (S18)$$

The first derivative can be simplified as $\frac{\partial(\rho E_\varphi)}{\partial \rho} = E_\varphi + \rho\frac{\partial E_\varphi}{\partial \rho}$, and the total force becomes

$$\langle \boldsymbol{F} \rangle = \frac{1}{2}\Re\mathrm{e}\left[-\varepsilon_\mathrm{b}\alpha^* \frac{|E_\varphi|^2}{\rho}\boldsymbol{n_\rho} + \varepsilon_\mathrm{b}\alpha^* E_\varphi^* \frac{1}{\rho}\left(E_\varphi + \rho\frac{\partial E_\varphi}{\partial \rho}\right)\boldsymbol{n_\rho} + \varepsilon_\mathrm{b}\alpha^* E_\varphi^* \frac{1}{\rho}\frac{\partial(\rho E_\varphi)}{\partial z}\boldsymbol{n_z}\right] \quad (S19)$$

which simplifies to

$$\langle \boldsymbol{F} \rangle = \frac{1}{2}\Re\mathrm{e}\left[-\varepsilon_\mathrm{b}\alpha^* \frac{|E_\varphi|^2}{\rho}\boldsymbol{n_\rho} + \varepsilon_\mathrm{b}\alpha^* \frac{|E_\varphi|^2}{\rho}\boldsymbol{n_\rho} + \varepsilon_\mathrm{b}\alpha^* \frac{\partial E_\varphi}{\partial \rho}\boldsymbol{n_\rho} + \varepsilon_\mathrm{b}\alpha^* E_\varphi^* \frac{1}{\rho}\frac{\partial(\rho E_\varphi)}{\partial z}\boldsymbol{n_z}\right] = \frac{1}{2}\Re\mathrm{e}\left[\varepsilon_\mathrm{b}\alpha^* E_\varphi^* \left(\frac{\partial E_\varphi}{\partial \rho}\boldsymbol{n_\rho} + \frac{1}{\rho}\frac{\partial(\rho E_\varphi)}{\partial z}\right)\boldsymbol{n_z}\right] \quad (S20)$$

which is **equation 3** in the main text. Further simplification can be achieved by computing the necessary derivatives:

$$\frac{\partial(\rho E_\varphi)}{\partial z} = -\rho E_0 e^{ik_0 \cos\theta_0 z} \frac{J_1(k_0 \sin\theta_0 \,\rho)}{\sin\theta_0} k_0 \cos\theta_0 \quad (S21a)$$

$$\frac{\partial E_\varphi}{\partial \rho} = iE_0 e^{ik_0 \cos\theta_0 z} \frac{J_1'(k_0 \sin\theta_0 \,\rho)}{\sin\theta_0} k_0 \sin\theta_0 = ik_0 E_0 e^{ik_0 \cos\theta_0 z} J_1'(k_0 \sin\theta_0 \,\rho) \quad (S21b)$$

where we have exploited the recurrence relation of Bessel's functions: $J_m'(x) = \frac{1}{2}[J_{m-1}(x) - J_{m+1}(x)]$. For $m = 0, J_0'(x) = \frac{1}{2}[J_{-1}(x) - J_1(x)] = \frac{1}{2}[-J_1(x) - J_1(x)] = -J_1(x)$. Inserting these expressions together with E_φ yields the final expression:

$$\langle \boldsymbol{F} \rangle = \frac{1}{2}\Re\mathrm{e}\left[\varepsilon_\mathrm{b}\alpha^* E_0^2 \left(\frac{k_0}{\sin\theta_0} J_1(x_\rho) J_1'(x_\rho)\boldsymbol{n_\rho} + i\frac{k_0 \cos\theta_0}{\sin^2\theta_0} J_1^2(x_\rho)\boldsymbol{n_z}\right)\right] \quad (S22)$$

which is **equation 4** in the main text, with $x_\rho = k_0 \sin\theta_0 \,\rho$. As stated there, the negative sign of the imaginary part of the conjugate polarizability α^* will yield a positive longitudinal force. Interestingly, the $\frac{\cos\theta_0}{\sin^2\theta_0}$ means that this longitudinal force will be greater for small-angle Bessel beams, which resemble plane waves. On the other hand, when $\theta_0 \to \frac{\pi}{2}$, the longitudinal force approaches zero.

S5. CDA method for optical force calculations in plasmonic rod dimers

We follow the method outlined in [14], although we note the use of SI units below. Each of the rods in the dimer is modeled as a point dipole $\boldsymbol{p_i}$ characterized by a polarizability α_i. Under this approximation, the optical response of a dimer in a medium with dielectric constant $\varepsilon_b = \varepsilon_0 n_b^2$ can be obtained by solving a system of equations:

$$\begin{aligned} \boldsymbol{p_1} &= \varepsilon_b \alpha_1 \boldsymbol{E_1} = \varepsilon_b \alpha_1 \left(\boldsymbol{E_{inc,1}} - \overline{\boldsymbol{A_{12}}} \cdot \boldsymbol{p_2}\right) \\ \boldsymbol{p_2} &= \varepsilon_b \alpha_2 \boldsymbol{E_2} = \varepsilon_b \alpha_2 \left(\boldsymbol{E_{inc,2}} - \overline{\boldsymbol{A_{21}}} \cdot \boldsymbol{p_1}\right) \end{aligned} \qquad (S23)$$

where $\boldsymbol{E_i}$ corresponds to the total electric field at the position of dipole $\boldsymbol{p_i}$, $\boldsymbol{E_{inc,1}}$ is the incident field on that position and the product $\overline{\boldsymbol{A_{ij}}} \cdot \boldsymbol{p_j}$ gives the electric field caused by dipole $\boldsymbol{p_j}$ at the position of dipole $\boldsymbol{p_i}$. $\overline{\boldsymbol{A_{ij}}}$ is a second order tensor given by

$$\overline{\boldsymbol{A_{ij}}}(\boldsymbol{r_{ij}}) = \frac{e^{ikr_{ij}}}{4\pi\varepsilon_b r_{ij}} \left[k^2 \left(\boldsymbol{n_{ij}} \otimes \boldsymbol{n_{ij}} - \bar{\boldsymbol{I}}\,\right) + \frac{ikr_{ij} - 1}{r_{ij}^2} \left(3\boldsymbol{n_{ij}} \otimes \boldsymbol{n_{ij}} - \bar{\boldsymbol{I}}\,\right) \right] \qquad (S24)$$

where $\boldsymbol{r_{ij}}$ is the position vector separating the dipoles. $r_{ij} = |\boldsymbol{r_{ij}}|$, $\boldsymbol{n_{ij}} = \frac{\boldsymbol{r_{ij}}}{r_{ij}}$ and $\bar{\boldsymbol{I}}$ is the identity dyadic. The symbol $\otimes$ denotes the dyadic product. For two dipoles, the system S11 can be easily solved as

$$\begin{aligned} \boldsymbol{p_1} &= \frac{\varepsilon_b \alpha_1 \left(\boldsymbol{E_{inc,1}} - \varepsilon_b \alpha_2 \, \overline{\boldsymbol{A_{12}}} \cdot \boldsymbol{E_{inc,2}}\right)}{\bar{\boldsymbol{I}} - \varepsilon_b^2 \, \alpha_1 \alpha_2 \, \overline{\boldsymbol{A_{12}}} \cdot \overline{\boldsymbol{A_{21}}}} \\ \boldsymbol{p_2} &= \frac{\varepsilon_0 \varepsilon_b \alpha_2 \left(\boldsymbol{E_{inc,2}} - \varepsilon_b \alpha_1 \, \overline{\boldsymbol{A_{21}}} \cdot \boldsymbol{E_{inc,1}}\right)}{\bar{\boldsymbol{I}} - \varepsilon_b^2 \, \alpha_1 \alpha_2 \, \overline{\boldsymbol{A_{21}}} \cdot \overline{\boldsymbol{A_{12}}}} \end{aligned} \qquad (S25)$$

where it must be noted that the fraction here corresponds to a right-matrix division. With the expressions for both dipole moments, the total electric field at any point in space can be calculated as

$$\boldsymbol{E}(\boldsymbol{r}) = \boldsymbol{E_{inc}}(\boldsymbol{r}) + \bar{\boldsymbol{A}}(\boldsymbol{r} - \boldsymbol{r_1}) \cdot \boldsymbol{p_1} + \bar{\boldsymbol{A}}(\boldsymbol{r} - \boldsymbol{r_2}) \cdot \boldsymbol{p_2} \qquad (S26)$$

where $\boldsymbol{r_1}$ and $\boldsymbol{r_2}$ correspond to the position vectors of the two dipoles and the corresponding magnetic field can be obtained as $\boldsymbol{B}(\boldsymbol{r}) = \frac{1}{i\omega} \boldsymbol{\nabla} \times \boldsymbol{E}(\boldsymbol{r})$. Once the total field is obtained, the optical force on dipole $\boldsymbol{p_i}$ can be calculated by invoking the Lorentz Force equation [12], [13], [15]:

$$\boldsymbol{F_i} = \Re\mathrm{e}(\boldsymbol{p_i} \cdot \boldsymbol{\nabla_i}) \Re\mathrm{e}(\boldsymbol{E_i}) + \Re\mathrm{e}\left(\frac{d\boldsymbol{p_i}}{dt}\right) \times \Re\mathrm{e}(\boldsymbol{B_i}) \qquad (S27)$$

which for harmonic fields, can be time-averaged as

$$\langle \boldsymbol{F_i} \rangle = \Re\mathrm{e}[(\boldsymbol{p_i^*} \cdot \boldsymbol{\nabla_i}) E_i + i\omega \boldsymbol{p_i^*} \times \boldsymbol{B_i}] \qquad (S28)$$

where * denotes the complex conjugate. For incident plane waves, this expression can be simplified to the sum of the contributions of the incident light on the individual dipoles plus an interaction term, both of which require calculating the field across all the simulation space (**equation S17**) and the computationally costly gradients in **equation S19** [13]. However, the complex Bessel beam requires full calculation using **equation S19**.

Formally speaking, approximation of nanoparticles as dipoles with polarizabilities α_i derives from the quasi-static approximation for spheres. However, this approximation can be extended to ellipsoidal objects and other geometries. For gold nanorods, we follow the expression derived by Kuwata et al. [16]:

$$\alpha \approx \frac{V}{\left(\Gamma + \frac{\varepsilon_b}{\varepsilon - \varepsilon_b}\right) + A_x(\Gamma)(n_b x)^2 + B_x(\Gamma)(n_b x)^4 - i \frac{4\pi^2 n_b^3}{3} \frac{V}{\lambda_0^3}} \qquad (S29)$$

where V is the rod volume, λ_0 is the incident wavelength and $x = \frac{\pi a}{\lambda_0}$ is the size parameter, with a being the particle size. $A_x(\Gamma)$ and $B_x(\Gamma)$ are functions dependent on the geometrical depolarization factor Γ. For rods, this factor is given by

$$\Gamma_{rod} = \frac{\left[(\xi - 1)^3 - 2 - (\xi - 2\xi - 1)\sqrt{\xi^2 - 2\xi + 2}\right]}{3(\xi - 1)^3} \qquad (S30)$$

where ξ is the aspect ratio between the long and short dimensions of the rod. The functions $A_x(\Gamma)$ and $B_x(\Gamma)$ are then

$$A_x(\Gamma) = -0.4865\Gamma - 1.046\Gamma^2 + 0.8481\Gamma^3$$
$$B_x(\Gamma) = 0.019\Gamma + 0.1999\Gamma^2 + 0.6077\Gamma^3 \quad (S31)$$

An important parameter to consider is interparticle distance, since for closely positioned dipoles electrostatic contributions to the induced fields, neglected under the dipolar approximation, become relevant. This effect can be accounted for by other methods, such as the use of Mie theory or more complex DDA approaches. To elucidate the impact that this may have on our calculations, we compare our CDA approach to full wave FDTD results in the case of a linearly y-polarized plane wave with intensity 0.4 W/μm^2 intensity plane wave incident on the analyzed dimers, with a separation distance of 100 nm. This comparison is displayed in **Figure S4a**, and it can be seen that the overall shape of both the optical force and the extinction cross-section (calculated using the extinction theorem as shown in [17]) is quite similar.

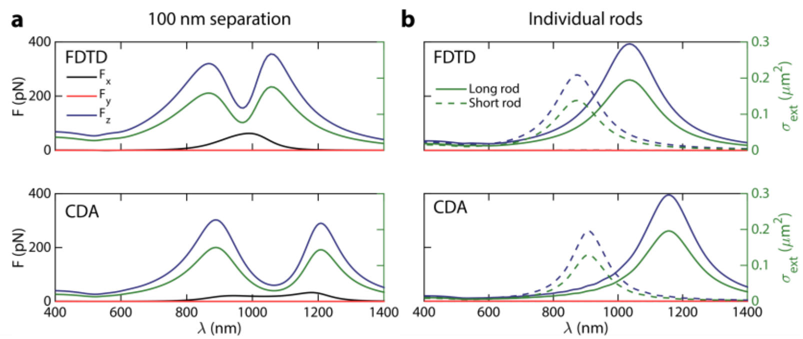

Fig. S4. a) Comparison of FDTD and CDA solutions for a dimer with a 100 nm interparticle distance illuminated by a y-polarized plane wave with intensity 0.4 W/μm^2, similarly to Figure 1d in the main text. b) FDTD and CDA results for the individual components of the dimer (distinguished by solid/dashed lines). The background refractive index for all calculations is set to $n_b = 1.5$.

The main difference between the CDA and FDTD methods is the location of the extinction peak corresponding to the larger rod in the dimer. While the FDTD peaks are located at 860 and 1050 nm, respectively; the CDA peaks are located at 880 and 1200 nm. The big redshift between the second peak locations between the methods mainly affects the longitudinal force, which closely follows the extinction cross-section, although the bigger gap between resonances in the CDA method flattens the transverse x-component force. Other than that, quantitative values do not change very significantly, with both longitudinal forces peaking around 300 pN.

While the interparticle distance might play a role in these discrepancies, the rod's polarizability modeling in **equation S20** can highly influence the spectral locations of the individual resonances. **Figure S4b** shows the results of both methods for the individual rods. Here, the spectral positions of the peaks closely match the dimer results, with the CDA methods having a redshifted second peak with respect to FDTD simulations. Here, the FDTD peaks are located at 870 and 1035 nm, while the CDA peaks are at 905 and 1150 nm. This means that the polarizability modelling in **equation S20** is the main responsible for the discrepancy of results, rather than the interparticle distance. It must be highlighted that the overall trends of forces and extinctions are similar between methods, meaning that the CDA method's precision is enough for our purpose in this work.

S6. Transversal forces on plasmonic dimers

As seen in the previous sections, the optical force by an azimuthally polarized Bessel beam on a plasmonic nanoparticle will have a positive longitudinal component as well as a radial component. In a plasmonic dimer, the interaction between particles, driven by the coupling tensors $\overline{\boldsymbol{A}_{ij}}$, might show important deviations from this behaviour. To check this, we provide in Figure S5 the missing components from **Figure 2e-f**, F_x and F_y. As in the CDA simulations the dimer was displaced from the center of the beam in the -x direction, $F_x = -F_\rho$ and $F_y = F_\varphi$ in the cylindrical coordinate system.

As expected, the longitudinal force, when the dimer is parallel with polarization, is accompanied by a strong x-component force, corresponding to a radial direction. In contrast, the y-component is near zero, as is expected of the azimuthal component for a single plasmonic particle.

On the other hand, when dimers are placed perpendicularly to the azimuthal polarization, as shown in the solid lines, all the components are near-zero, supporting the fact that independent control of the different degrees of freedom of the nanomotor can be achieved by orienting the dimers in this direction.

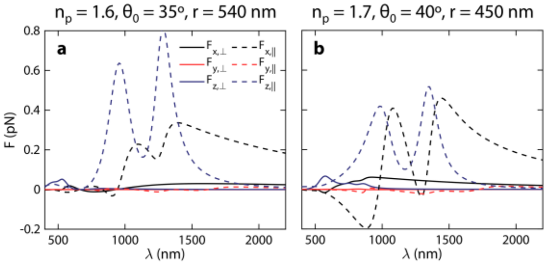

Fig. S5. Optical forces on the highlighted cases from **Figure 2e** (a) and **Figure 2f** (b) with all components F_x, F_y and F_z. Solid lines represent a perpendicular orientation to polarization, and dashed lines a parallel orientation with respect to polarization.

Some other relevant features can be found here. As shown in **equation 4** in the main text, the longitudinal force is proportional to $\frac{\cos\theta_0}{\sin^2\theta_0}$, meaning that lower angles, as in **Figure S5a** with respect to **Figure S5b**, will generally have larger longitudinal forces. Regarding the radial force, its peaks are redshifted with respect to the longitudinal peaks, and the radial force has a remarkably weaker decay for longer wavelengths. This can be attributed to the fact that the radial force depends on the real part of the polarizability rather than the imaginary part. This prompts a different behavior, including the sign flip found at ~900 nm in **Figure S5b**.

Furthermore, in resonance, the force is positive (drawing the dimer to the center of the beam), which points to the dimer being located at a point where the product $J_1(x_\rho) \cdot J'_1(x_\rho)$ is negative. Since both functions are positive for small x_ρ, this suggests the opportunity to place the dimers closer to the center to avoid the radial force altogether. However, smaller distances between dimers might induce further coupling between elements, significantly altering the overall optical response.

S7. Longitudinal forces and torques on nanomotors

For plane wave illumination, the longitudinal components of force and torque, not shown in **Figure 3** in the main text, are depicted in **Figure S6**. It can be seen again that the longer nanomotor, bigger in size and containing more dimers, is characterized by larger forces and torques. The F_z component is much larger than either $F_{||}$ or $F_\perp$ in **Figure 3**, meaning that any plane wave illumination for lateral movement is going to be associated with a strong pushing force. Furthermore, the pushing force is significant for all considered wavelengths, which allows to control pushing movement independently of lateral movement if out-of-resonance wavelengths are employed (as, for example, $\lambda = 1700$ nm). The longitudinal torque T_z, on the contrary, supposes a minor contribution against the $T_{||}$ component in **Figure 3**.

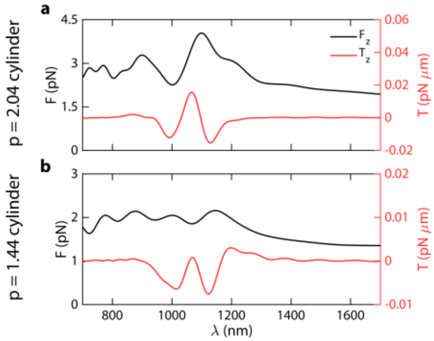

Fig. S6. Longitudinal forces and torques F_z and T_z exerted by a linearly polarized plane wave with intensity 0.4 mW/ μm² on the a) $p = 2.04$ and b) $p = 1.44$ aspect ratio nanomotors as a function of wavelength.

The longitudinal component torques for both nanomotors are shown in **Figure S7**. The magnitude of both torques is much lower than that of the dominant component T_y, especially in the case of the longer cylinder. This further allows the one-dimensional treatment employed in the diffusion simulations in the main text.

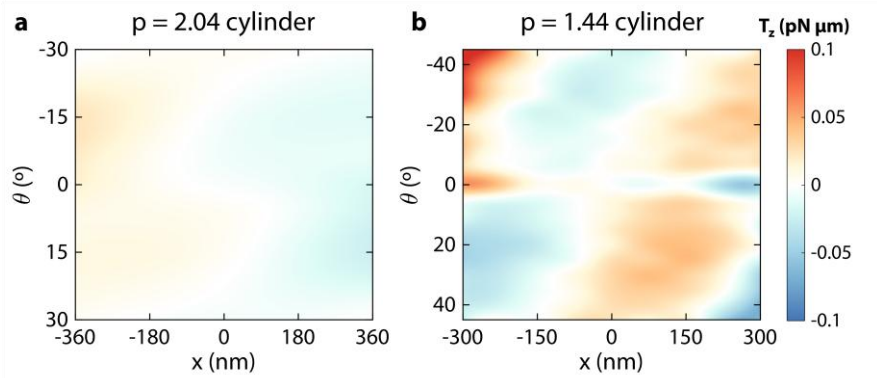

Fig. S7. Longitudinal torque T_z exerted on the a) $p = 2.04$ and b) $p = 1.44$ aspect ratio nanomotors for different positions and orientations.

S8. Extension of the scheme for controlled rotations

The proposed scheme in the main text is designed to only provide translational lateral motion. However, for non-absorbing structures, a helicity-dependent optical torque T_z can be exerted using circularly polarized light [18], [19]. Furthermore, as shown by Käll and coworkers [20], plasmonic nanorods can efficiently transduce the spin angular momentum from circularly polarized light into a rotatory motion, something that has been important to many different applications. In particular, plasmonic rods could be fitted into the design, providing a helicity-dependent rotatory motion that can be made independent from other lateral motions if said rods are tuned to a different illumination wavelength, i.e., a wavelength below 1000 nm, where scattering from the gold nanorod dimer becomes important.

Although absorption can play a role into the torque induced on such nanorods, the most important contribution comes from scattering processes, for which the time-averaged torque T_z can be expressed as [21]

$$T_z \propto \Delta\alpha^2 \qquad (S32)$$

where $\Delta\alpha = \alpha_x - \alpha_y$ the polarizability anisotropy of the nanorods. As seen in this equation, longer rods, characterized by larger anisotropies, will allow higher torques. On the other hand, larger aspect ratios in plasmonic nanorods lead to a redshift of their plasmonic resonance [22]. We account for the necessary torque that these nanomotors require to overcome Brownian motion:

$$T_B = \sqrt{2\gamma_r k_B T} = \sqrt{k_B T \frac{64}{3} \pi \eta_W a^3} \qquad (S33)$$

where $\gamma_r = \frac{32}{3}\pi\eta_W a^3$ is the rotational friction coefficient for a cylinder with radius a [23]. For the $p = 1.44$ design, the Brownian torque threshold is approximately 0.02 pN µm, which is relatively low (although higher than the T_z achieved with linearly polarized illumination). Thus, rods with relatively low anisotropies can be employed for this purpose. To further separate the response of these rods from the translational dimers, we employ silver, whose resonances are typically found at shorter wavelengths [22], as the material of choice for these rods.

The proposed design is illustrated in **Figure S8a**. Silver nanorods (100 nm long, 50 nm wide and thick) are inserted between the spaces left by the four gold nanorod dimers. Their radial position is the same as the gold nanorod dimers (450 nm from the center of the $p = 1.44$ cylinder) and their orientation is perpendicular to the azimuthal orientation of the Bessel beam to preserve optical pulling. For the $p = 1.44$ cylinder nanomotor and appropriate Bessel beam illumination (40º, 1300 nm wavelegth), the forces and torques suffer almost negligible changes upon introduction of such silver nanorods, as demonstrated in **Figure S8b**. In particular, the optical pulling force is reduced by 5% approximately.

Figure S8c-d shows the transversal forces exerted on the $p = 1.44$ nanomotor by a circularly polarized wave, with and without the silver nanorods. Transversal forces have a similar pattern to their linearly polarized counterparts in **Figure 3** of the main text. However, as power is distributed between both *x* and *y* polarizations, both F_x and F_y have significant contributions above zero, although lower than their linearly polarized counterparts. This does not change much upon introduction of the silver nanorods, whose resonances are at shorter wavelengths. It is important to highlight that helicity changes just produce a permutation of the forces, as the nanomotor is not geometrically chiral.

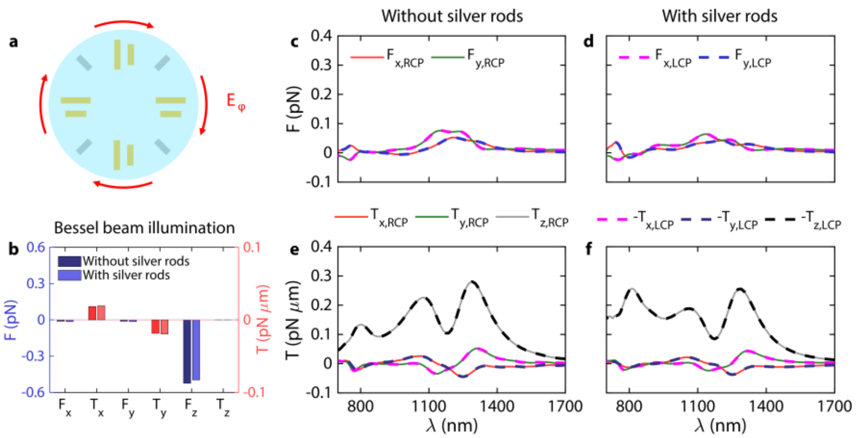

Fig. S8. a) Scheme of the 3D nanomotor design, to which four silver nanorods have been added in the available space between the dimers (the center of the rods, as the dimers, is 450 nm away from the cylinder center). As with the dimers, the silver nanorods are perpendicularly oriented to the azimuthal polarization of a pulling Bessel beam. b) Comparison between the different force and torque components between the $p = 1.44$ nanomotor without and with the silver nanorods for the corresponding Bessel beam illumination. c) Optical transversal forces exerted by a 0.4 mW µm^{-2} circularly polarized plane wave on the $p = 1.44$ nanomotor without silver rods and (d) with them. Solid and dashed lines represent the different helicity (RCP or LCP) of the incoming wave. e) Optical torque exerted by the same circularly polarized plane wave on the $p = 1.44$ nanomotor without silver rods and (f) with them. Note that the LCP torque components, represented by dashed lines, have undergone a sign change to facilitate comparison with their RCP counterparts.

On the other hand, the torques, shown in **Figure S8e-f** have a very dominant T_z component, which was not found for linear polarization (as shown in **Figure S6b**). Two peaks are found at 110 and 1300 nm, following the two resonances of the gold nanorods. These, however, are coincident with peak values of F_x, F_y, T_x and T_y, meaning that T_z cannot be independently controlled with these resonances. A third, weaker T_z peak appears for a 800 nm wavelength, which is not correlated with strong forces or torques, and thus, usable for independent rotation control. This peak can be associated with the small lateral forces found at the same wavelength here and in **Figure S5b**.

Upon introduction of the silver nanorods, their contribution significantly enhances this weaker T_z peak (around 92%), without significantly altering any of the other transversal forces and torques. Again, as the system is not chiral, the torques do not change in magnitude upon helicity changes. However, the transversal components are again interchanged, and all torques components undergo a sign flip. Thus, a helicity-dependent optical torque one order of magnitude above the Brownian threshold can be applied independently from other forces and torques, allowing efficient motion control over clockwise and anticlockwise rotations.

S9. Characterization of the hydrodynamic properties of cylinders

The hydrodynamic behavior of cylinders has been the subject of intense research, following its frequent appearance in colloids, such as the tobacco mosaic virus or short DNA fragments [24], [25], [26]. Most works trying to describe these hydrodynamic properties have focused on long aspect-ratio ("rod-like") cylinders [27]. However, the aspect ratios of the cylinders described in this work are rather short, with aspect ratios $p \lesssim 2$. Therefore, the more general formulae developed by Ortega and García de la Torre are followed [28]. In this work, the diffusion tensor component $D_r^{\perp}$ is related to a rotational time τ_a by

$$\tau_a = \frac{1}{6D_r^{\perp}} \qquad (S34)$$

For aspect ratios $p > 0.75$, as is the case in this work, τ_a can be calculated from the interpolating equation

$$\frac{\tau_a}{\tau_0} = 1.18 + 1.116(\ln p + 0.2877)^2 - 0.2417(\ln p + 0.2877)^3 + 0.4954(\ln p + 0.2877)^4 \qquad (S35)$$

where $p = L/D$ is the length-to-diameter aspect ratio and $\tau_0 = \frac{\pi L^3 \eta_W}{4p^2 k_B T}$. Here, $\eta_W = 10^{-3}$ Pa·s is the water viscosity, k_B is Boltzmann constant and T is the temperature (taken as 300 K). On the other hand, the translational diffusion coefficient D_t is given by

$$D_t = \frac{k_B T}{f_t} \qquad (S36)$$

related to a translational friction coefficient f_t, given by a similar expression:

$$\frac{f_t}{f_{t0}} = 1.009 + 1.395 \cdot 10^{-2}(\ln p) + 7.880 \cdot 10^{-2}(\ln p)^2 + 6.040 \cdot 10^{-3}(\ln p)^3 \qquad (S37)$$

with $f_{t0} = 6\pi\eta_W L\left(\frac{3}{16p^2}\right)^{1/3}$.

References

[1] S. Ramo, J. R. Whinnery, and T. Van Duzer, *Fields and waves in communication electronics*, 3rd edition. John Wiley & Sons, Inc., 1994. Accessed: Apr. 29, 2025. [Online]. Available: https://www.wiley.com/en-us/Fields+and+Waves+in+Communication+Electronics%2C+3rd+Edition-p-9780471585510

[2] X. Li, J. Chen, Z. Lin, and J. Ng, "Optical pulling at macroscopic distances," *Sci Adv*, vol. 5, no. 3, p. eeau7814, 2019, doi: 10.1126/sciadv.aau7814.

[3] W. J. Tomlinson and R. E. Wagner, "Coupling efficiency of optics in single-mode fiber components," *Appl Opt*, vol. 21, no. 15, pp. 2671–2688, Aug. 1982, doi: 10.1364/AO.21.002671.

[4] B. E. A. Saleh and M. Carl. Teich, *Fundamentals of photonics*, 3rd edition. Wiley, 2019.

[5] C. F. Bohren and D. R. Huffman, *Absorption and Scattering of Light by Small Particles*, 1st edition. Wiley, 1983.

[6] S. Sukhov and A. Dogariu, "Negative nonconservative forces: Optical 'tractor Beams' for arbitrary objects," *Phys Rev Lett*, vol. 107, no. 20, p. 203602, Nov. 2011, doi: 10.1103/PHYSREVLETT.107.203602/FIGURES/4/THUMB-NAIL.

[7] A. Jannasch, A. F. Demirörs, P. D. J. Van Oostrum, A. Van Blaaderen, and E. Schäffer, "Nanonewton optical force trap employing anti-reflection coated, high-refractive-index titania microspheres," *Nat Photonics*, vol. 6, no. 7, pp. 469–476, Jul. 2012, doi: 10.1038/NPHOTON.2012.140;SUBJMETA=1107,1110,624,639;KWRD=OPTICAL+MA-NIPULATION+AND+TWEEZERS.

[8] Z. Wu, Y. Han, J. Wang, and Z. Cui, "Generation of Bessel beam sources in FDTD," *Opt Express*, vol. 26, no. 22, p. 28727, Oct. 2018, doi: 10.1364/oe.26.028727.

[9] L. Novotny and B. Hecht., *Principles of Nano-Optics*, 2nd Edition. Cambridge University Press, 2012.

[10] J. Chen, P. Wang, Z. Lin, and J. Ng, "Analytical partial wave expansion of vector Bessel beam and its application to optical binding," *Opt Lett*, vol. 35, no. 10, pp. 1674–1676, May 2010, doi: 10.1364/OL.35.001674.

[11] B. Liang, M. Bai, H. Ma, N. Ou, and J. Miao, "Wideband analysis of periodic structures at oblique incidence by material independent FDTD algorithm," *IEEE Trans Antennas Propag*, vol. 62, no. 1, pp. 354–360, 2014, doi: 10.1109/TAP.2013.2287896.

[12] L. Novotny, "Forces in Optical Near-Fields," *Near-Field Optics and Surface Plasmon Polaritons*, pp. 123–141, Aug. 2001, doi: 10.1007/3-540-44552-8_7.

[13] A. G. Hoekstra, M. Frijlink, L. B. F. M. Waters, and P. M. A. Sloot, "Radiation forces in the discrete-dipole approximation," *Journal of the Optical Society of America A*, vol. 18, no. 8, pp. 1944–1953, 2001.

[14] V. D. Miljković, T. Pakizeh, B. Sepulveda, P. Johansson, and M. Käll, "Optical forces in plasmonic nanoparticle dimers," *Journal of Physical Chemistry C*, vol. 114, no. 16, pp. 7472–7479, Apr. 2010, doi: 10.1021/jp911371r.

[15] J. P. Gordon, “Radiation Forces and Momenta in Dielectric Media,” *Phys Rev A (Coll Park)*, vol. 8, no. 1, p. 14, Jul. 1973, doi: 10.1103/PhysRevA.8.14.

[16] H. Kuwata, H. Tamaru, K. Esumi, and K. Miyano, “Resonant light scattering from metal nanoparticles: Practical analysis beyond Rayleigh approximation,” *Appl Phys Lett*, vol. 83, no. 22, pp. 4625–4627, Dec. 2003, doi: 10.1063/1.1630351.

[17] G. W. Mulholland, C. F. Bohren, and K. A. Fuller, “Light Scattering by Agglomerates: Coupled Electric and Magnetic Dipole Method,” *Langmuir*, vol. 10, pp. 2533–2546, 1994.

[18] P. L. Marston and J. H. Crichton, “Radiation torque on a sphere caused by a circularly-polarized electromagnetic wave,” *Phys Rev A (Coll Park)*, vol. 30, no. 5, p. 2508, Nov. 1984, doi: 10.1103/PhysRevA.30.2508.

[19] P. H. Jones, O. M. Maragò, and G. Volpe, *Optical Tweezers: Principles and Applications*, 1st ed. Cambridge: Cambridge University Press, 2015.

[20] L. Tong, V. D. Miljković, and M. Käll, “Alignment, rotation, and spinning of single plasmonic nanoparticles and nanowires using polarization dependent optical forces,” *Nano Lett*, vol. 10, no. 1, pp. 268–273, Jan. 2010, doi: 10.1021/NL9034434/SUPPL_FILE/NL9034434_SI_006.AVI.

[21] L. Shao, Z. J. Yang, D. Andrén, P. Johansson, and M. Käll, “Gold Nanorod Rotary Motors Driven by Resonant Light Scattering,” *ACS Nano*, vol. 9, no. 12, pp. 12542–12551, Nov. 2015, doi: 10.1021/ACSNANO.5B06311/ASSET/IMAGES/LARGE/NN-2015-063118_0003.JPEG.

[22] G. Baffou, “Thermoplasmonics: Heating metal nanoparticles using light,” *Thermoplasmonics: Heating Metal Nanoparticles Using Light*, pp. 1–291, Oct. 2017, doi: 10.1017/9781108289801.

[23] M. Liu, T. Zentgraf, Y. Liu, G. Bartal, and X. Zhang, “Light-driven nanoscale plasmonic motors,” *Nat Nanotechnol*, vol. 5, no. 8, pp. 570–573, Jul. 2010, doi: 10.1038/nnano.2010.128.

[24] J. K. G. Dhont, *An introduction to dynamics of colloids*, 2nd edition. Elsevier, 2003.

[25] G. F. Bonifacio, T. Brown, G. L. Conn, and A. N. Lane, “Comparison of the electrophoretic and hydrodynamic properties of DNA and RNA oligonucleotide duplexes,” *Biophys J*, vol. 73, no. 3, pp. 1532–1538, 1997, doi: 10.1016/S0006-3495(97)78185-2.

[26] N. C. Santos and M. A. R. B. Castanho, “Teaching light scattering spectroscopy: The dimension and shape of tobacco mosaic virus,” *Biophys J*, vol. 71, no. 3, pp. 1641–1650, Sep. 1996, doi: 10.1016/S0006-3495(96)79369-4.

[27] M. Mercedes Tirado, C. López Martínez, and J. García De La Torre, “Comparison of theories for the translational and rotational diffusion coefficients of rod-like macromolecules. Application to short DNA fragments,” *J Chem Phys*, vol. 81, no. 4, pp. 2047–2052, Aug. 1984, doi: 10.1063/1.447827.

[28] A. Ortega and J. Garcia De La Torre, “Hydrodynamic properties of rodlike and disklike particles in dilute solution,” *J Chem Phys*, vol. 119, no. 18, pp. 9914–9919, Nov. 2003, doi: 10.1063/1.1615967.
